# Complicated cataract surgery: strong leadership protects patients

**Published:** 2019-02-10

**Authors:** John Buchan

**Affiliations:** 1 Ophthalmologist: International Centre for Eye Health, London School of Hygiene and Tropical Medicine, London, UK.

It is said that “safety is no accident.” This means that safe medical practice does not happen by chance. Careful planning of your operating environment will protect patients, but it requires a holistic approach as surgeons are only as good as the environment they work in and the team that supports them.

## Stock take and procurement

A well-led team will be doing regular stock takes: identifying when stocks are running low and ordering more supplies before the shelf is empty. This is essential if a complication arises and an alternative IOL is needed or a piece of equipment is urgently required (such as an automated vitrector, iris hooks or a certain suture).

## Sterilisation schedules

Standard surgical instruments may be arranged in trays appropriate to the operation being performed. When an additional or unexpected instrument is needed, can it be provided at very short notice? This might require single wrapped items to be held in store, or a tray of additional possible instruments to be sterilised at the start of the theatre list and accessed as needed. Whatever the system, it requires good leadership to ensure it is set up in order to make the surgeon's life as easy as possible when the situation is complex.

## Clinical pathways to success

Much has been written about how checklists and protocols reduce human error and protect patients. For example, the protocol at the start of the surgical day could include discussing patients on the theatre list with potentially difficult cataract operations and then verifying that the equipment is ready and the full operating team is prepared and appropriately trained in any procedures that may be needed.

If you are in a team where other people put patients on the operating list for you, you may only see the patient on the day of surgery. Consider creating a checklist to guide the history taking and eye examination, both of which are essential in order to identify cataract patients with other pre-existing conditions.

**Figure:**
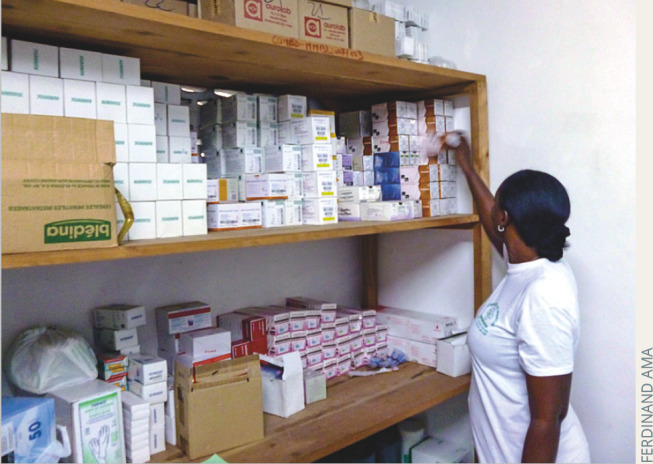
Regular stock-taking is good practice.

A history checklist might include:

- Any history of serious ocular trauma
- Previous eye surgery or laser
- Diabetic status

The examination checklist could include:

- Endothelial guttae
- Pseudoexfoliation
- Extent of dilation
- Eye pressure

## Teaching trainees to handle complicated surgery

If you are involved in teaching other surgeons, complicated cases provide excellent opportunities for training. Trainees can be involved in pre-operative planning and discussion of strategies. The next time they encounter the same scenario, they may no longer be a trainee.

